# Nurse Farms, Hospitals, and Private Nursing Institutions

**Published:** 1887-03-19

**Authors:** 


### Nurse Farms, Hospitals, and Private Nursing Institutions.

### MISS MANSON'S REPLY.

I have to thank Mrs. Bedingfeld for the opportunity she has given me for further stating my views on a subject which I consider it decidedly to the advantage of the public to be freely discussed. I should, however, first like to state that my paper on "Private Nursing Institutions" was read before the Hospitals Association, and reprinted in the The Hospital on the 19th February; a full report of the discussion thereon, which was throughout without one dissentient voice, in its favour, appeared on the 26th February—but it was not until the 5th March that I had the pleasure of reading Mrs. Bedingfeld's criticisms.

Mrs. Bedingfeld commences her attack by stating that some of my remarks "are not only incorrect but also unfair." All incorrect remarks are necessarily unfair, and all strictures can only be unfair in so far as they are incorrect, and that my paper contained incorrect statements I beg distinctly to contradict. The first statement in my paper that is considered incorrect or unfair—Mrs. Bedingfeld does not say which—is "that institutions farm out nurses for their own profit." I wish to put only one question to Mrs. Bedingfeld with regard to that sentence. May I ask for what philanthropic object these institutions exist, if not for the benefit of their promoters? or to what charity the surplus of her own institution is devoted? Should hospitals make any profit from their private nursing home it benefits the hospital, and so certainly, though indirectly, benefits the nurses attached to that hospital, as well as the sick within its walls.

It is also stated that "it is not correct that some of the institutions engage women who have no previous experience of nursing." Here Mrs. Bedingfeld only half quoted my words, for I concluded, "who are sent for six months' or one year's promiscuous training at some hospital and are there advertised as 'hospital-trained nurses.'" So-called "trainees" are admitted for this purpose for periods ranging from six to twelve months, at several hospitals, notably at "Guy's" and the "London"; and, as a sister at the last-named hospital, I have myself had such a "trainee" in my ward who was picking up as much medical and surgical nursing knowledge as could be condensed into six months, before becoming attached to one of the oldest private nursing institutions—the "Nursing Sisters' Institution," 4, Devonshire Square. When I was first appointed Matron of St. Bartholomew's, women were admitted there also on the same terms for the benefit of the same institution.

Mrs. Bedingfeld herself states with emphasis that, as superintendent of a private nursing institution, she requires "that a nurse should have at least *one* year's complete training." I am happy to hear it; it is, as I have proved, more than some of her colleagues do. Mrs. Bedingfeld can, however, have but little practical experience as to what is a necessary training before a nurse can be described as *completely* trained, and in consequence deserving of the confidence of the public on that ground, or she must be aware that the members of the medical profession and the committees attached to at least three of our largest and most thorough nursing schools in London—St. Bartholomew's, St. Thomas's, and Guy's—have fixed the complete term of training at a period of not less than three years, and that it is universally acknowledged that in no case can a woman be considered trained in a shorter period than two years of varied experience.

Mrs. Bedingfeld further remarks that "a nurse's training is far from complete in many ways when she leaves the hospital." That, I should say, depends a good deal on where Mrs. Bedingfeld's nurses gain their experience—and I may add that I think it is hardly fair to criticise a woman's capabilities as a trained nurse after only *one* year's experience. If Mrs. Bedingfeld will refer to my paper, she will see that I emphatically state, that hospital authorities cannot be too careful in their choice of women for *private* nursing, stating that "many women who make excellent ward nurses are unsuitable for private nursing." I further say, that at the close of their training the hospital authorities should satisfy themselves that the nurse is suitable in "character and manners," as well as in her nursing qualifications, for private work. I think a careful selection can be much more easily made in a large hospital, where there are plenty to choose from, than in an institution where applications are limited. I also think that Mrs. Bedingfeld must be misinformed when she writes—"that there are more *good* nurses than there is work for"; and "that nurses holding first-class certificates have been almost starving for want of employment." I can only say that the demand made upon me for private parish and district nurses is far in excess of what I am able to supply, and that the majority of our great infirmaries are in need of, the inmates in sore need of, thoroughly trained nurses.

I repeat, that a *washing uniform* for a nurse is indispensable from a sanitary point of view. The patient referred to, whose opinion Mrs. Bedingfeld thinks worth quoting, "who likes a nurse to look as unlike a nurse as possible," can hardly have had a fair opportunity of forming a satisfactory opinion of what a nurse's appearance should be. A visit to No. 13, West Smithfield, and the sight of our uniforms—dainty, speckless, and eminently becoming—might convert him from the error of his ways.

As to mental attendants, it is hardly necessary to state that they cannot be classed in the same category with sick-nurses. The attendant's disguise often simply forms a part of the treatment for allaying the patient's suspicions, just as the medical man himself is sometimes compelled to make his calls under the name of a private friend.

I have only to add, and I gladly avail myself of the opportunity of doing so, that I consider the whole system of private nursing institutions, as they now exist, however well managed they may be—and I do not hesitate to affirm that some of them are well managed—to be based on a *false principle*.

Private nurses form such an important branch of the nursing profession, that the system which places them under the immediate and *sole* control of a *private individual*, who is not necessarily interested in raising the standard of nursing as a profession, but who rather tends to regard nurses as a means whereby to gain an income, is lowering to the profession of nursing as a whole.

Though primarily directed against the evils arising from this wrong principle of private nursing institutions, my paper was likewise intended to point out to hospitals their respon ibility in the matter towards the wealthier public, and also towards the nurses themselves, in the manner in which they should be protected and cared for. I look forward to the day, not very far distant, when private nursing institutions will cease to exist, and be supplanted by thoroughly organised Homes, for supplying the public with trained and certificated nurses attached to all our large metropolitan and county hospitals. St. Bartholomew's, St. Thomas's, Guy's, the London, St. George's, Middlesex and Westminster, should make it their aim to have on their private nursing staff from one to two hundred nurses. The "mutual accommodation" system would then become unnecessary, and I venture to remark that the nursing profession, as a whole, would, in conjunction with the public, be materially benefited. From a financial point of view, it should be the object of all hospitals to direct the channel of whatever funds can be legitimately made by the profession of nursing towards the support of hospitals, and in consequence towards the support of the nursing profession. This can be partly done by adopting the suggestion made by me, of starting a private paying hospital in connection with every first-rate medical and nursing school, and by largely increasing the number of nurses employed on their private nursing staff. To make the scheme a thorough success, the Homes must be thoroughly well appointed and managed on a liberal scale. It is very important that after a stated term of service the nurses should be awarded their own earnings, paying a small sum per case to the Home, and for board and lodging when unemployed. I believe this plan has been generously inaugurated by the London Association of Nurses, 62, New Bond Street. Part of the surplus money earned by the nurses should be invested for their benefit in a pension fund, so that during ill-health and old age they might be awarded an annuity at a certain ratio, according to length of service. To this fund they might also be encouraged to contribute certain specified sums yearly, receiving in return an additional pension.

My thanks are due to Miss Meyrick for the courtesy of tone in which her letter is couched,—a virtue which is conspicuous by its absence in some of the criticisms upon my paper. I regret that there is one point upon which we do not agree. The Editor, in his marginal note, has anticipated my chief reason for advocating the adoption of an outdoor uniform to be worn by nurses, when attached to a private or district nursing staff.

The tone of "A Trained Nurse's" view is so rude that I can take no notice of her article, for I am quite unqualified to descend to the level of her argument.

I may remark briefly, by way of answer to the criticisms of the 12th instant, that the letter of a "Barrister and Private Patient,"—who, from the drift of his remarks, is presumably within measurable distance of the "Woolsack," and whose opinions are diametrically opposed to my own,—has failed to convince me that my suggestions for placing private nursing on a thoroughly professional basis is either unwise or impracticable. Time will show!

The opinion of a "Lady Superintendent of a Provincial Nursing Institution" would possibly have been of more value had she read my paper!

"A Private Nurse," speaking for private nurses generally—I presume, with their concurrence,—challenges my article. It is evident from her letter that she is attached to the London Association of Nurses. "A Private Nurse" first objects to my statement, that "institutions which make any stipulation as to length of time a nurse must have been trained, are few and far between." I will simply inform her of two facts in connection with the institution of which she is a member, and of which she is probably in ignorance. On the 1st of August 1886 a nurse on the staff of the London Association of Nurses was received at St. Bartholomew's Hospital as a special probationer, paying her own expenses of a guinea a week for three months' experience in surgical nursing, explaining to me "that she did not feel *justified* in undertaking the charge of surgical and operation cases, as she had only been trained for a short period at a special hospital." Further, on the 1st of February 1887, a nurse, who had been working in this hospital for two years, was received *direct* as a member of the above-mentioned institution, without any letter of inquiry concerning her work or character having been addressed to either myself or the office. I agree with "A Private Nurse," that the terms of the London Association "being liberal," highly efficient nurses join its ranks sometimes. Amongst them are six trained in our own nursing school, of whose excellent nursing qualifications I am well aware. But I must add that they are the *only nurses* considered by me thoroughly satisfactory, who have during my six years' superintendence joined a private nursing institution.

I repeat that *at present* the *best* nurses are still retained on the regular nursing staff of the hospitals in positions ranging from Lady Superintendent to Staff nurses. When I state that 48 of our certificated nurses have during the given time (six years) continued to work in one or other of these positions, it will be seen that the comparison is *not* in favour of private nursing, and that I make my statement, as I invariably do, *from personal experience*.

### PROFESSOR SPENCE AND SUICIDE.

A CHARACTERISTIC story is told which well illustrates the pawky humour of the late Professor Spence. Being examined in court concerning the death of a gentleman when there was a suspicion of suicide, the Professor dwelt perhaps somewhat tediously on an obstruction to the flow of the blood from the left side of the heart. One of the counsel for the suicide theory interrupted him a little impatiently, saying: "Come, come, Professor; you know we are long past that." "Yes," quietly rejoined the Professor, "I daresay *you* are, but the blood wasn't."

